# The Survival Benefit of Chemoradiotherapy following Induction Chemotherapy with Gemcitabine Plus Nab-Paclitaxel for Unresectable Locally Advanced Pancreatic Cancer

**DOI:** 10.3390/cancers13184733

**Published:** 2021-09-21

**Authors:** Ryoji Takada, Kenji Ikezawa, Kazuma Daiku, Shingo Maeda, Yutaro Abe, Makiko Urabe, Yugo Kai, Takuo Yamai, Nobuyasu Fukutake, Tasuku Nakabori, Hiroyuki Uehara, Reiko Ashida, Hirofumi Akita, Hidenori Takahashi, Teruki Teshima, Kazuyoshi Ohkawa

**Affiliations:** 1 Department of Hepatobiliary and Pancreatic Oncology, Osaka International Cancer Institute, Osaka 541-8567, Japan; ryoji.takada@oici.jp (R.T.); kazuma.daiku@oici.jp (K.D.); shingo.maeda@oici.jp (S.M.); abe-yu@mc.pref.osaka.jp (Y.A.); makiko.urabe@oici.jp (M.U.); yugo.kai@oici.jp (Y.K.); takuo.yamai@oici.jp (T.Y.); fukutake.nobuyasu.du@mail.hosp.go.jp (N.F.); tasuku.nakabori@oici.jp (T.N.); uehara@oici.jp (H.U.); kazuyoshi.ohkawa@oici.jp (K.O.); 2 Department of Cancer Survey and Gastrointestinal Oncology, Osaka International Cancer Institute, Osaka 541-8567, Japan; rashida@wakayama-med.ac.jp; 3 Department of Surgery, Osaka International Cancer Institute, Osaka 541-8567, Japan; hirofumi.akita@oici.jp (H.A.); htakahashi8@gesurg.med.osaka-u.ac.jp (H.T.); 4 Department of Radiation Oncology, Osaka International Cancer Institute, Osaka 541-8567, Japan; t.teshima@osaka-himak.or.jp

**Keywords:** pancreatic ductal adenocarcinoma, chemoradiation, induction chemotherapy, conversion surgery, combination chemotherapy, local response

## Abstract

**Simple Summary:**

An optimal therapeutic strategy for unresectable locally advanced pancreatic cancer (UR-LAPC) has not been established. The aim of this retrospective study was to evaluate the therapeutic efficacy, progression-free survival (PFS), and overall survival (OS) of patients with UR-LAPC who underwent gemcitabine plus nab-paclitaxel (GnP) as first-line chemotherapy followed by chemoradiotherapy (CRT) compared to chemotherapy alone (CTx) at our department in a Japanese cancer referral center between February 2015 and July 2018. CRT resulted in significantly better PFS and OS than CTx. In the multivariate analyses, CRT following induction chemotherapy was identified as an independent prognostic factor for OS. In summary, patients with UR-LAPC experienced favorable treatment outcomes after receiving GnP as the first-line chemotherapy, especially when receiving additional CRT after tailored courses of induction chemotherapy. Thus, this treatment strategy represents a promising treatment option for selected patients with UR-LAPC.

**Abstract:**

An optimal therapeutic strategy for unresectable locally advanced pancreatic cancer (UR-LAPC) has not been established. This study investigated the therapeutic efficacy of chemoradiotherapy (CRT) following induction chemotherapy with gemcitabine plus nab-paclitaxel (GnP) (CRT group) compared with systemic chemotherapy alone (CTx group) in patients with UR-LAPC. This was a retrospective study of 63 consecutive patients with UR-LAPC treated at our department in a Japanese cancer referral center between February 2015 and July 2018. We excluded patients who underwent other regimens and those enrolled in another prospective study. The CRT group (*n* = 25) exhibited significantly better progression-free survival (PFS) and overall survival (OS) than the CTx group (*n* = 20, PFS 17.9 vs. 7.6 months, *p* = 0.044; OS 29.2 vs. 17.4 months, *p* < 0.001). In the multivariate analyses, CRT following induction chemotherapy was identified as an independent prognostic factor for OS. Seven (15.6%) patients underwent conversion surgery, all of whom were in the CRT group. The R0 resection rate was 85.7% (6/7). In summary, patients with UR-LAPC experienced favorable treatment outcomes after receiving GnP as the first-line chemotherapy, especially when receiving additional CRT. Thus, this treatment strategy represents a promising treatment option for selected patients with UR-LAPC.

## 1. Introduction

Pancreatic cancer (PC) has one of the worst prognoses among diseases worldwide [1,2] despite recent progress in chemotherapy, which has improved the survival of patients with PC [3,4,5,6]. Among cases of locally advanced PC, borderline resectable PC and unresectable locally advanced PC (UR-LAPC) are anatomically defined according to the degree of major vessel involvement, including the celiac artery (CA), the superior mesenteric artery (SMA), the portal vein (PV), and the superior mesenteric vein (SMV) [7]. UR-LAPC accounts for 10–20% of PC cases [8,9], and is considered unresectable even in the absence of metastatic disease due to its high involvement with nearby structures. According to National Comprehensive Cancer Network (NCCN) guidelines, systemic chemotherapy is generally recommended for patients with UR-LAPC to prolong overall survival (OS) and improve quality of life [7]. Chemoradiotherapy (CRT), or stereotactic body radiation therapy, is proposed to be performed with induction chemotherapy or in select patients who are not candidates for combination chemotherapy (e.g., gemcitabine plus nab-paclitaxel [GnP] and FOLFIRINOX). CRT could prevent or delay local progression and improve medium- to long-term survival rates in patients with UR-LAPC [10,11]. However, despite two randomized controlled trials (RCTs), the effect of upfront CRT on OS in patients with UR-LAPC remains controversial [11,12].

The purpose of induction chemotherapy, which is performed prior to CRT, is to gain the full benefit of locoregional therapy by selecting patients without occult metastasis and those who have early tumor progression [13]. Although induction chemotherapy using gemcitabine or gemcitabine plus erlotinib did not show a survival benefit in previous RCTs [10,14], it is possible that more intensive induction chemotherapy including GnP and FOLFIRINOX could improve treatment outcomes, as these regimens are more efficient in suppressing occult metastasis and tumor growth [3,15,16]. However, there are few reports concerning induction chemotherapy with GnP in patients with UR-LAPC [17]. Therefore, this study aimed to compare therapeutic efficacy, progression-free survival (PFS), and OS between patients with UR-LAPC treated with CRT following induction chemotherapy with GnP (CRT group) and those who received systemic chemotherapy alone (CTx group). We also examined the prognostic factors associated with OS in patients with UR-LAPC.

## 2. Materials and Methods

**Figure 1:**
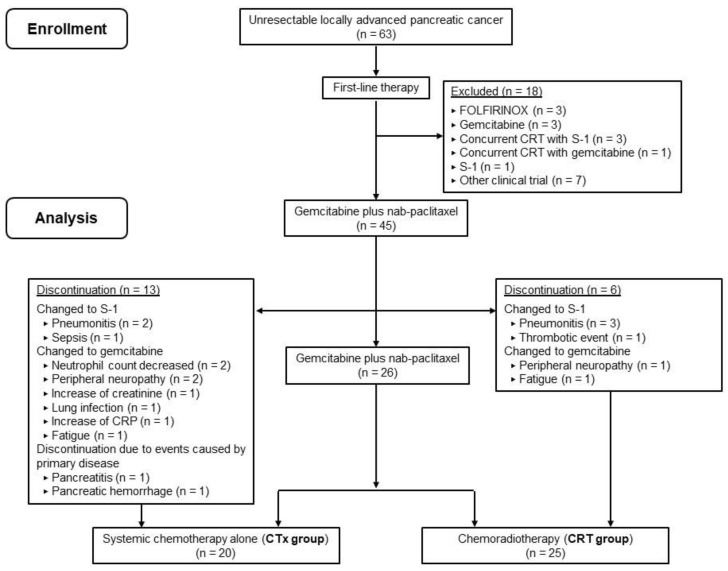
Consort flow diagram of the treatment strategy for unresectable locally advanced pancreatic cancer in our department.

We retrospectively reviewed data from 63 consecutive patients with pathologically proven UR-LAPC who received chemotherapy or CRT at our department at the Osaka International Cancer Institute between February 2015 and July 2018. UR-LAPC was defined as no apparent distant metastasis except for regional lymph node metastasis, tumor contact with the CA or SMA > 180°, tumor contact with the aorta, surgically unresectable tumor with common hepatic artery and/or gastroduodenal artery involvement, or surgically unresectable tumor with PV/SMV involvement according to NCCN guidelines [7]. The clinical staging and resectability of all tumors were assessed by a board of cancer specialists comprising surgeons, radiologists, and oncologists. Exclusion criteria were patients who had received other first-line treatment regimens (*n* = 11) and those enrolled in another prospective study for patients with UR-LAPC (*n* = 7, Figure 1). After excluding these patients, 45 patients who received GnP as first-line chemotherapy were included in our analysis. We collected data for each patient regarding age, sex, Eastern Cooperative Oncology Group (ECOG) performance status (PS), primary tumor location, duodenal invasion, and biliary drainage, along with carbohydrate antigen 19-9 (CA19-9) and carcinoembryonic antigen (CEA) levels.

### 2.1. Treatment

The GnP regimen comprised gemcitabine (1000 mg/m^2^) and nab-paclitaxel (125 mg/m^2^), which was administered weekly for 3 weeks, followed by a 1-week rest period. The treatment cycle was repeated every 4 weeks. Dosages of each drug and treatment schedule were adjusted by the physicians based on the occurrence of adverse events (AEs), patient comorbidities, and the patient’s condition. Consenting patients received CRT based on their comorbidities and clinical conditions, and if they met the following criteria: a stable or better response to induction chemotherapy, potential resectability, no appearance of metastasis, no direct invasion to the gastrointestinal wall, and a not-overly-large tumor size.

We used two kinds of radiation protocol: the UR-LAPC setting and the preoperative setting. The purpose of the UR-LAPC setting was local control of the tumor. In this setting, CRT was administered at a total radiation dose of 50.4 Gy/25 fractions/5 weeks with effective photon energies of ≥6 MV. CRT was targeted to the primary pancreatic tumor using computed tomography-based, three-dimensional conformal radiation therapy (3D-CRT) or intensity-modulated radiotherapy (IMRT)/volumetric modulated arc therapy (VMAT). The preoperative setting was used for patients who were scheduled to undergo conversion surgery. In this setting, CRT was targeted not only to the primary pancreatic tumor, but also to the perivascular and retroperitoneal tissues for preventing local recurrence after surgery. CRT targeted to the primary pancreatic tumor was administered at a total radiation dose of 50 Gy/25 fractions/5 weeks, whereas CRT targeted to the CA/SMA, retroperitoneal soft tissue, and para-aortic region was administered at 60 Gy/25 fractions/5 weeks [18] with a simultaneous integrated boost. During CRT, single-agent chemotherapy with gemcitabine or S-1 (an oral fixed-dose combination of three active drugs; tegafur (oral prodrug of fluorouracil), gimeracil, and oteracil) was administered as a radiosensitizer. A dose of gemcitabine equivalent to the last dose administered during the induction chemotherapy term was also provided, and S-1 was administered at a dose of 40 mg/m^2^ twice daily on the day of irradiation.

### 2.2. Evaluation of Treatment Outcomes and Adverse Events

Hematological and non-hematological AEs were graded in accordance with the Common Terminology Criteria for Adverse Events version 4.0. Tumor response was assessed in accordance with the revised Response Evaluation Criteria in Solid Tumors guidelines (version 1.1). The best response from the initiation of first-line GnP therapy to disease progression was assessed. OS was defined as the period from the initiation of first-line chemotherapy (GnP) to the date of death from any cause. PFS was defined as the period from the initiation of first-line GnP to the date of disease progression. Regarding PFS, transfer to other hospitals was defined as censored, while discontinuation of first-line GnP due to AEs and patient refusal was not defined as censored. The duration of the local response was defined as the period from the initiation of first-line GnP to the day of progression at the primary site. Regarding the duration of the local response, conversion surgery (CS), transfer to other hospitals, and death without primary site progression were defined as censored. Time-to-treatment failure (TTF) was defined as the period from the initiation of first-line GnP to the day of discontinuation from any cause, including disease progression and treatment toxicity. Regarding TTF, transfer to other hospitals and change of treatment policy to CRT were defined as censored. Data from patients who were alive at the end of the follow-up period (November 2020) were regarded as censored.

### 2.3. Statistical Analysis

We compared baseline characteristics between the groups using a Mann–Whitney U-test for continuous variables and chi-square or Fisher’s exact tests for categorical variables. Analyses of PFS, OS, duration of the local response, and TTF were performed using the Kaplan–Meier method, and differences were evaluated using a log-rank test. Univariate and multivariate analyses were performed using a Cox proportional hazards model to identify significant prognostic factors associated with OS. Hazard ratios (HRs) and 95% confidence intervals (CIs) were calculated. Factors with a *p*-value < 0.05 in the univariate analysis were entered into the multivariate Cox models. Statistical analyses were performed using EZR (Saitama Medical Center, Jichi Medical University, Saitama, Japan), a graphical interface for the R Commander software package for Windows (version 1.53) [19]. Statistical significance was set at *p* < 0.05.

## 3. Results

### 3.1. Patient Characteristics

Patient characteristics are summarized in Table 1. The median age was 68 (range, 48–82) years, and 24 (53.3%) patients were men. The ECOG PS was 0 in 33 (73.3%) patients and one in 12 (26.7%) patients. Primary tumors were located at the pancreatic head in 31 (68.9%) patients and at the pancreatic body/tail in 14 (31.1%) patients. The median tumor size was 37 mm (range, 20–100 mm). Eleven (24.4%) patients had duodenal invasion due to PC. Fifteen (33.3%) patients underwent endoscopic biliary drainage for biliary obstruction due to PC.

Of the 45 patients who received GnP as the first-line chemotherapy for UR-LAPC, 25 (55.6%) received chemotherapy followed by CRT (CRT group), whereas 20 (44.4%) received systemic chemotherapy only (CTx group). A comparison of patient characteristics between the CRT and CTx groups is shown in Table 1. There were no significant differences in age, sex, ECOG PS, tumor location, tumor size, and biliary drainage, or in CA 19-9 and CEA levels between the two groups. In contrast, the rate of duodenal invasion was significantly higher in the CTx group than in the CRT group (CRT group, 12.0%; CTx group, 40.0%; *p* = 0.041). Three patients with duodenal invasion in the CRT group successfully underwent CRT because they had experienced tumor shrinkage and disappearance of the duodenal invasion with induction GnP therapy.

### 3.2. Treatment Outcomes and AEs

The overall response rate (ORR) was 40.0% (18/45; complete response, *n* = 1; partial response, *n* = 7; Table 2). There were no significant differences in ORR between the CRT and CTx groups (CRT group, 48.0%; CTx group, 30.0%; *p* = 0.359). The disease control rate (DCR) was 93.3% (42/45); this was higher in the CRT group than in the CTx group, although the difference was not statistically significant (CRT group, 100.0%; CTx group, 85.0%; *p* = 0.080).

The median TTF in all patients was 6.8 months (95% CI 3.1–11.6 months). Nineteen (42.2%) patients could not continue first-line treatment with GnP (Figure 1). CTx was discontinued in 13 (65.0%) patients. The chemotherapeutic regimen changed in 11 patients from GnP to S-1 or gemcitabine due to GnP-related AEs (pneumonitis, *n* = 2; sepsis, *n* = 1; neutrophil count decrease, *n* = 2; peripheral neuropathy, *n* = 2; an increase in creatinine, *n* = 1; lung infection, *n* = 1; an increased CRP level, *n* = 1; and fatigue, *n* = 1). Two patients discontinued GnP due to primary disease events (pancreatitis, *n* = 1; pancreatic hemorrhage, *n* = 1). In the CRT group, the median time from initiation of GnP to CRT was 4.5 (interquartile range, 3.4–6.6) months. Six (24.0%) patients changed the chemotherapeutic regimen during induction chemotherapy from GnP to S-1 or gemcitabine due to GnP-related AEs (pneumonitis, *n* = 3; fatigue, *n* = 1; peripheral neuropathy, *n* = 1; and thromboembolic event, *n* = 1). The chemotherapeutic regimen administered during CRT was gemcitabine in 20 patients and S-1 in four patients, and one patient changed from gemcitabine to S-1 due to pneumonitis during CRT.

For the radiotherapy protocol, the UR-LAPC setting was used in 19 (76%) patients and the preoperative setting in six (24%) patients. The radiotherapy technique was 3D-CRT in 13 patients and IMRT/VMAT in 12 patients. All patients completed planned radiotherapy. Four patients experienced AEs associated with CRT (hemobilia, *n* = 2; duodenal ulcer, *n* = 1; gastritis, *n* = 1). After completion of CRT, 12 patients underwent GnP again, while six patients underwent treatment with gemcitabine, five patients underwent treatment with S-1, and one patient underwent modified FOLFIRINOX. One patient underwent CS without restarting chemotherapy after CRT.

Seven patients (15.6%) underwent CS, all of whom belonged to the CRT group. A summary of patients who underwent CS is shown in Table 3. The R0 resection rate was 85.7% (6/7). Six of the patients who underwent CS received adjuvant chemotherapy with S-1 monotherapy.

### 3.3. Survival and Disease Progression

**Figure 2:**
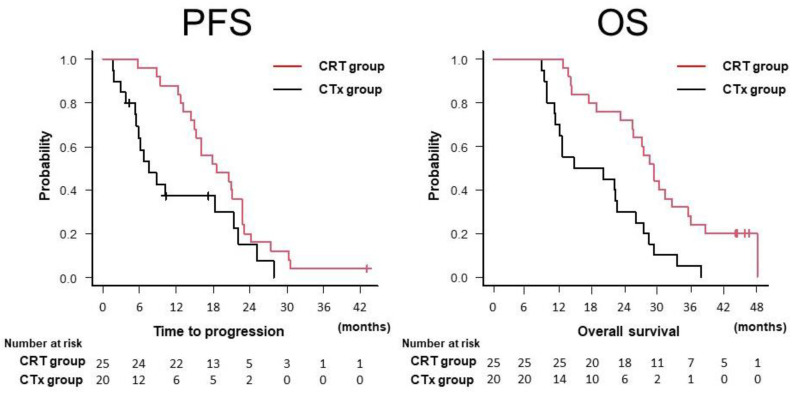
Kaplan–Meier curves for PFS and OS in the CRT and CTx groups. OS, overall survival; PFS, progression-free survival; The CRT group (*n* = 25) included patients who underwent chemoradiotherapy following induction chemotherapy with gemcitabine plus nab-paclitaxel. The CTx group (*n* = 20) included patients who underwent systemic chemotherapy alone.

**Figure 3:**
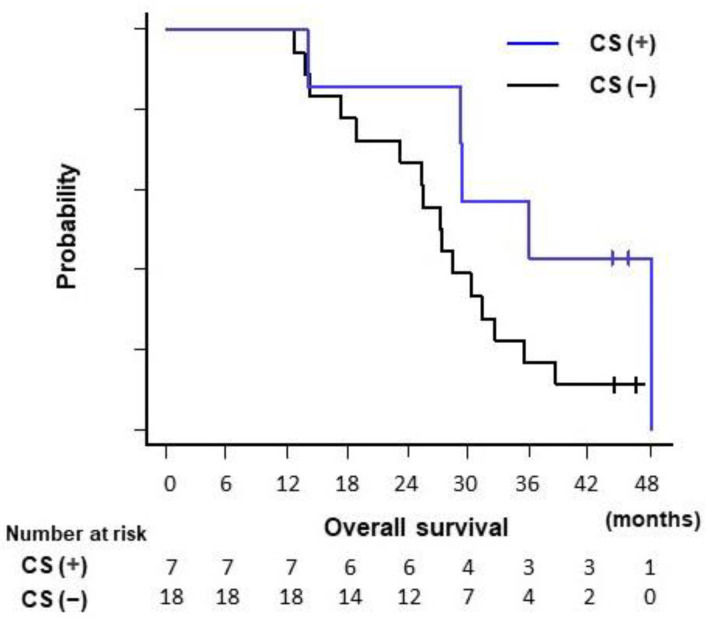
A comparison of overall survival between patients who underwent CS and those who did not among patients in the CRT group. CS, conversion surgery. The CRT group (*n* = 25) included patients who underwent chemoradiotherapy following induction chemotherapy with gemcitabine plus nab-paclitaxel.

The median duration of follow-up was 25.5 months (range, 8.9–48.2 months). Forty-one (91.1%) patients had died by the end of the follow-up period. The median PFS and OS for all patients were 15.2 and 25.5 months, respectively. The median PFS and OS were significantly longer in the CRT group than in the CTx group (PFS, 17.9 vs. 7.6 months, *p* = 0.044; OS, 29.2 vs. 17.4 months, *p* < 0.001; Table 2, Figure 2). The median duration of the local response was also significantly longer in the CRT group than in the CTx group (24.2 vs. 10.3 months, *p* = 0.005; Table 2). All seven patients who underwent CS experienced recurrence. Among the CRT group, the median OS was longer in patients who underwent CS (*n* = 7) than in those who did not (*n* = 18), although the difference was not statistically significant (36.0 vs. 27.2%, *p* = 0.09) (Figure 3).

We evaluated predictive factors associated with OS (Table 4). In the univariate analysis of ten variables, tumor size (≥40 mm) and CRT following induction chemotherapy were significantly associated with OS (HR 2.25, 95% CI 1.18–4.30; *p* = 0.014 and HR 0.34, 95% CI 0.18–0.65; *p* = 0.001, respectively). In the multivariate analysis using the two variables that had *p*-values < 0.05 in the univariate analysis, CRT following induction chemotherapy was identified as a statistically significant independent prognostic factor for OS (HR 0.40, 95% CI 0.21–0.79; *p* = 0.008).

## 4. Discussion

Although more intensive chemotherapy including GnP and FOLFIRINOX has been performed in patients with UR-LAPC, their median OS remains poor, ranging from 17 to 19 months in most studies [17,20,21]. Multidisciplinary strategies combining systemic and locoregional treatments are required to improve OS in these patients. In our current study of 45 patients with UR-LAPC who received GnP as first-line chemotherapy, the CRT group exhibited favorable PFS, OS, and duration of local response. Notably, the median OS in the CRT group was no less than 29.2 months, which is a remarkable result when compared to previous findings.

Although the efficacy of induction chemotherapy for UR-LAPC was not established in two previous RCTs (LAP07 and JCOG1106) [10,14], this may have been due to an insufficient intensity of induction chemotherapy, as those studies used gemcitabine or gemcitabine plus erlotinib as induction chemotherapy. The rates of tumor progression in the induction term were 23.1% for patients in LAP07 and 16.3% for patients in JCOG1106, while the respective median PFS rates were 9.9 months and 10.4 months. However, in our study, GnP, which is considered one of the most efficient chemotherapeutic regimens for advanced PC [3,16,22], was used as first-line chemotherapy for UR-LAPC. Among all patients enrolled in the study, the median PFS was 15.2 months, with a favorable DCR (93.3%). The CRT group exhibited favorable PFS and DCR when compared with the CTx group. In the multivariate analyses adjusted for patient characteristics, CRT following induction chemotherapy was identified as an independent prognostic factor for OS. There may have been some patient selection bias because patients with a good response to chemotherapy tended to undergo CRT; however, CRT following induction GnP chemotherapy could be a promising treatment strategy to improve OS with favorable local response. Further prospective studies are required to confirm the survival benefit of CRT following the induction of GnP chemotherapy.

Radiotherapy is inappropriate for UR-LAPC with duodenal invasion because it may cause duodenal toxicities such as bleeding, ulcers, and perforation [23,24]. However, in our study, three patients in the CRT group had duodenal invasion at GnP initiation. After confirming the disappearance of duodenal invasion due to tumor shrinkage, these patients successfully underwent CRT without RT-related AEs, suggesting that CRT can be a promising treatment for UR-LAPC with duodenal invasion if tumor shrinkage is achieved with induction chemotherapy.

CS for UR-LAPC has recently attracted attention because it may offer a chance of radical resection [25]. CS may be a treatment option for responders to chemotherapy and/or CRT with the expectation of a longer OS and treatment-free survival [26,27]. Previous studies have reported that multi-drug combination therapy improved the conversion rate (15–36%) [17,28]. In our study, CS was performed in seven (15.6%) patients. Prior to CS, six patients underwent CRT in the preoperative setting. The R0 resection rate was 85.7%, which was higher than that reported in a previous study of patients with UR-LAPC (41%) [17]. Although the prognostic difference was not statistically significant, the prognosis for patients who underwent CS was favorable (OS, 36.0 months) when compared with that of patients who did not. Further prospective studies are required to determine the survival benefits of CS.

This study had some limitations. First, this non-randomized, retrospective study was conducted at a single referral center. In the multivariate analyses that adjusted for baseline characteristics, which differed between the CRT and CTx groups, CRT following induction chemotherapy was identified as an independent factor associated with favorable OS. However, selection bias remains a concern because CRT tended to be administered to patients showing a favorable response to induction chemotherapy. Second, the duration from GnP initiation to CRT initiation differed in each patient because it was determined based on the extent of tumor shrinkage, the physician’s discretion, and patient consent. NCCN guidelines recommend a preferable duration of induction chemotherapy of 4–6 months. In this study, the median duration of induction chemotherapy was approximately four months, which could be considered reasonable. Third, we excluded patients with UR-LAPC who underwent regimens other than GnP from the analysis, because the number of patients was small. Further studies on induction chemotherapy with intensive chemotherapeutic regimens including FOLFIRINOX may help to further define the clinical impact of CRT following induction chemotherapy in patients with UR-LAPC.

## 5. Conclusions

In conclusion, we found that patients with UR-LAPC experienced favorable treatment outcomes after receiving GnP as the first-line chemotherapy, especially when receiving additional CRT after tailored courses of GnP chemotherapy induction. Thus, this treatment strategy represents a promising treatment option for selected patients with UR-LAPC. Further prospective studies are warranted to investigate the oncological benefit of a treatment strategy that included induction GnP and subsequent CRT for UR-LAPC patients, and to identify the subsets of patients who would likely benefit from the inclusion of CRT in the treatment sequence.

## Figures and Tables

**Table 1:** Characteristics of patients with UR-LAPC who underwent gemcitabine plus nab-paclitaxel as first-line chemotherapy.

	All	CRT Group	CTx Group	*p*-Value
	(*n* = 45)	(*n* = 25)	(*n* = 20)	
Age (years) *	68 (48–82)	67 (48–82)	68 (48–76)	0.514
Sex (male/female)	24/21	11/14	10/10	0.769
ECOG PS (0/1)	33/12	17/8	16/4	0.502
Location (head/body and tail)	31/14	9/16	5/15	0.525
Tumor size (mm) *	37.0 (20.0–100.0)	33.0 (20.0–100.0)	38.5 (24.0–74.0)	0.134
Duodenal invasion (yes/no)	11/34	3/22	8/12	0.041
Biliary drainage (yes/no)	15/30	9/16	6/14	0.757
CA19-9 (U/mL) *	372.0 (<2.0–13, 856.0)	372 (<2.0–13, 856.0)	464 (<2.0–13, 292.0)	0.404
CEA (ng/mL) *	3.2 (0.8–103.3)	2.9 (0.8–103.3)	4.35 (1.5–100.4)	0.17
Follow-up period (months) *	25.5 (8.9–48.2)			

CA19-9, carbohydrate antigen 19-9; CA125, cancer antigen 125; CEA, carcinoembryonic antigen; ECOG PS, Eastern Cooperative Oncology Performance Status; UR-LAPC, unresectable locally advanced pancreatic cancer. The CRT group (*n* = 25) included patients who underwent chemoradiotherapy following induction chemotherapy with gemcitabine plus nab-paclitaxel. The CTx group (*n* = 20) included patients who underwent systemic chemotherapy alone. * Statistical significance at *p* < 0.05.

**Table 2:** Treatment outcomes for patients with UR-LAPC who underwent gemcitabine plus nab-paclitaxel as first-line chemotherapy.

	All	CRT Group	CTx Group	*p*-Value
	(*n* = 45)	(*n* = 25)	(*n* = 20)	
Best response				
CR	1	1	0	
PR	17	11	6	
SD	24	13	11	
PD	2	0	2	
NE	1	0	1	
Best overall response rate (%)	40	48	30	0.359 ^†^
Disease control rate (%)	93.3	100	85	0.080 ^†^
Progression-free survival time (months ^§^ [95% CI])	16.0 (12.3–20.9)	18.5 (15.0–22.7)	7.6 (5.3–21.4)	0.036 ^‡^
Duration of local response (months, ^§^ [95% CI])	22.0 (18.3–25.4)	24.2 (20.3–30.4)	10.3 (5.3–25.2)	0.005 ^‡^
Overall survival time (months, ^§^ [95% CI])	25.5 (18.8–28.5)	29.2 (25.4–35.4)	17.4 (11.2–25.9)	<0.001 ^‡^

CI, confidence interval; CR, complete response; NE, not evaluated; PD, progressive disease; PR, partial response; SD, stable disease; UR-LAPC, unresectable locally advanced pancreatic cancer. The CRT group (*n* = 25) included patients who underwent chemoradiotherapy following induction chemotherapy with gemcitabine plus nab-paclitaxel. The CTx group (*n* = 20) included patients who underwent systemic chemotherapy alone. ^†^ Fisher’s exact test, ^‡^ Log-rank test, ^§^ Median Statistical significance at *p* < 0.05.

**Table 3:** Characteristics of patients who underwent conversion surgery.

No.	Sex	Age (y)	ECOGPS	Tumor Size (mm)	Tumor Location	Duodenal Invasion	Duration of Induction Chemotherapy (Months)	Time to Surgery (Months)	Operation	Resection Margin	DFS (Months)	OS (Months)
				(Initial → CRT)								
1	F	64	1	29 → 24	Head	No	10.9	13	PD	R0	17.7	48.2
												(dead)
2	F	66	0	26 → 23	BT	No	2.3	4.9	DP-CAR	R1	15.8	45.9
												(alive)
3	F	68	1	26 → 16	BT	No	3.5	6.1	PD	R0	17.2	44.3
												(alive)
4	M	73	0	29 → 19	Head	Yes	6.1	10.3	PD	R0	12.7	36.0
												(dead)
5	F	66	0	45 → 18	BT	No	1.9	13.6	DP-CAR	R0	9.4	29.4
												(dead)
6	M	64	1	23 → 22	Head	No	4.8	11.6	PD	R0	8.2	29.3
												(dead)

**Table 4:** Prognostic factors associated with overall survival.

		Univariate Analysis		Multivariate Analysis	
		HR (95% CI)	*p*-Value	HR (95% CI)	*p*-Value
Age (years)	<70	1			
	≥70	1.37 (0.72–2.58)	0.338		
Sex	Male	1			
	Female	0.90 (0.48–1.68)	0.746		
ECOG PS	0	1			
	1	1.01 (0.49–2.08)	0.976		
Location	Body and tail	1			
	Head	0.93 (0.47–1.83)	0.825		
Tumor size (mm)	<40	1		1	
	≥40	2.25 (1.18–4.30)	0.014	1.71 (0.87–3.35)	0.119
Duodenal invasion	No	1			
	Yes	1.37 (0.68–2.75)	0.379		
Biliary drainage	No	1			
	Yes	0.75 (0.38–1.47)	0.398		
CRT following induction chemotherapy	No	1		1	
	Yes	0.34 (0.18–0.65)	0.001	0.40 (0.21–0.79)	0.008
CA19-9 (U/mL)	<1000	1			
	≥1000	1.53 (0.79–3.00)	0.21		
CEA (ng/mL)	<5.0	1			
	≥5.0	0.88 (0.45–1.72)	0.709		

CA125, cancer antigen 125; CA19-9, carbohydrate antigen 19-9; CEA, carcinoembryonic antigen; CI, confidence interval; CRT, chemoradiotherapy; ECOG PS, Eastern Cooperative Oncology Group Performance Status; HR, hazard ratio; OS, overall survival. Statistical significance at *p* < 0.05.

## Data Availability

The data presented in this study are available on request from the corresponding author. The data are not publicly available due to privacy issues.
